# 

**Published:** 1874-11

**Authors:** 


### CONTENTS:

### COMMUNICATIONS:

What does Experience Teach to be the best Material for filling Teeth? .... 449
Necrosis—A strange Case .... 456
Have smooth-faced Pluggers any advantage over Serrated? .... 458
Address delivered before the Aacademy of Dental Science 460
Synopsis of the Discussions of the American Dental Society of Europe .... 472
Correspondence .... 477

### EDITORIAL:

Lacto-Phosphate of Lime .... 480
Merrimack Valley Dental Association .... 483
Baxter's Automatic Mallet .... 484
A Law to regulate the Practice of Dentistry in the State of New Jersey .... 485
A Manual of Dental Mechanics .... 487
Ohio State Dental Society .... 488
Attention .... 490
Dental Convention .... 490
A new Hand Piece .... 491
Bars and Excavators .... 492
The History of Dentistry .... 492

